# Integrative Analysis of DNA Methylation Data and Transcriptome Data Identified a DNA Methylation-Dysregulated Four-LncRNA Signature for Predicting Prognosis in Head and Neck Squamous Cell Carcinoma

**DOI:** 10.3389/fcell.2021.666349

**Published:** 2021-04-01

**Authors:** Qiuxu Wang, Weiwei Yang, Wei Peng, Xuemei Qian, Minghui Zhang, Tianzhen Wang

**Affiliations:** ^1^Department of Stomatology, The First Affiliated Hospital of Shenzhen University Health Science Center, Shenzhen Second People’s Hospital, Shenzhen, China; ^2^Department of Stomatology, The Affiliated Zhongshan Hospital of Dalian University, Dalian, China; ^3^Department of Pathology, Harbin Medical University, Harbin, China; ^4^Department of Oncology, Chifeng City Hospital, Chifeng, China

**Keywords:** head and neck squamous cell carcinoma, DNA methylation, long-coding RNAs, biomarker, signature

## Abstract

Increasing evidence has demonstrated the crosstalk between DNA epigenetic alterations and aberrant expression of long non-coding RNAs (lncRNAs) during carcinogenesis. However, epigenetically dysregulated lncRNAs and their functional and clinical roles in Head and Neck Squamous Cell Carcinoma (HNSCC) are still not explored. In this study, we performed an integrative analysis of DNA methylation data and transcriptome data and identified a DNA methylation-dysregulated four-lncRNA signature (DNAMeFourLncSig) from 596 DNA methylation-dysregulated lncRNAs using a machine-learning-based feature selection method, which classified the patients of the discovery cohort into two risk groups with significantly different survival including overall survival, disease-specific survival, and progression-free survival. Then the DNAMeFourLncSig was implemented to another two HNSCC patient cohorts and showed similar prognostic values in both. Results from multivariable Cox regression analysis revealed that the DNAMeFourLncSig might be an independent prognostic factor. Furthermore, the DNAMeFourLncSig was substantially correlated with the complete response rate of chemotherapy and may predict chemotherapy response. Functional *in silico* analysis found that DNAMeFourLncSig-related mRNAs were mainly enriched in cell differentiation, tissue development and immune-related pathways. Overall, our study will improve our understanding of underlying transcriptional and epigenetic mechanisms in HNSCC carcinogenesis and provided a new potential biomarker for the prognosis of patients with HNSCC.

## Introduction

Head and neck squamous cell carcinoma (HNSCC) represents a heterogeneous group of malignancies derived from the oral cavity, larynx and pharynx, and has become the sixth most common cancer worldwide (Johnson et al., 2020). HNSCC is an aggressive, life-threatening disease associated with low survival rates because of the failure of early diagnosis. Although multimodal treatment, including surgery and chemoradiotherapy, is generally used in clinical practice, HNSCC patients after diagnosis still experienced low survival rates because of regional and distant metastatic spreading and insufficient effectiveness of therapeutic modalities (Dahiya and Dhankhar, 2016). Therefore, biomarkers have become essential tools to provide critically useful and cost-effective information for improving diagnosis and prognosis.

Long non-coding RNAs (lncRNAs) are the primary type of non-coding RNAs (ncRNAs). They have been widely reported to be involved in various biological progress through epigenetic, transcriptional, and post-transcriptional regulation via crosstalk with other RNA species or proteins (Perry and Ulitsky, 2016; Marchese et al., 2017). Increasing evidence and studies have demonstrated the widespread dysregulation of lncRNAs in many human diseases, including cancers (Fang and Fullwood, 2016). These dysregulated lncRNAs play essential roles in cancer development, progression, metastasis and therapy, have widely been recognized as potential attractive biomarkers and therapeutic targets (Jiang M.C. et al., 2019; Bao et al., 2020; Sun et al., 2020; Yan et al., 2020; Zhou et al., 2020). Recent studies found that aberrant expression of lncRNAs could be caused by altered DNA methylation contributing to carcinogenesis. The relationship between epigenetic alterations and lncRNAs expression has been revealed in several cancers (Hadji et al., 2016; Li et al., 2019; Zhang et al., 2019; Hou et al., 2020; Zhang et al., 2020). However, epigenetically dysregulated lncRNAs and their functional and clinical roles in Head and Neck Squamous Cell Carcinoma (HNSCC) are still not explored.

In this study, we performed an integrative analysis of DNA methylation data and transcriptome data to explore the relationship between epigenetic alterations and lncRNAs expression, as well as their prognostic value in HNSCC.

## Materials and Methods

### HNSCC Datasets

DNA methylation data (Illumina 27k methylation array), RNA-seq data and clinical data of 528 HNSC tumor tissues and 50 normal tissues were derived from the UCSC Xena Browser^1^. The DNA methylation data were preprocessed using the R package ‘‘RnBeads’’ as follows: (i) Removed SNP-enriched probes resulting in10131 probes with the last three bases of their sequences overlap with SNPs were removed; (ii) removed probes with missing values in more than 10% samples. (iii) Imputation was performed by calculating the median methylation level of each sample across all CpG sites and replacing all missing values for this sample at an individual CpG site with the median across all CpGs in the sample. Imputation replaced a median of 2 missing values per sample by estimation. Finally, a total of 392,302 probes of 578 samples were retained for further analysis. lncRNA expression profiles were obtained from RNA-seq data based on the GENCODE annotations^2^.

### Identification of DNA Methylation-Dysregulated lncRNA Biomarkers

Differential CpG site methylation between HNSC tumor tissues and normal tissues was identified using the R package “limma,” and those with FDR adjusted *p* < 0.05 and absolute mean methylation difference > 0.4 were considered as differentially methylated CpG sites. Then we investigated the association between lncRNA and differentially methylated CpG sites by calculating the Pearson correlation coefficient (PCC) between lncRNA expression and methylation levels of differentially methylated CpG sites. Those lncRNAs significantly correlated with differentially methylated CpG sites with | r| > 0.4 and *p* < 0.01 were considered as DNA methylation-dysregulated lncRNAs. The univariate and multivariate Cox regression analyses were performed to evaluate the association of DNA methylation-dysregulated lncRNAs and overall survival, and those DNA methylation-dysregulated lncRNAs significantly associated with overall survival were considered as DNA methylation-dysregulated lncRNA biomarkers.

### Development of DNA Methylation-Dysregulated lncRNA Signature (DNAMeLncSig)

We used the stepwise regression method by successively adding or removing variables for DNA methylation-dysregulated lncRNA biomarkers to identify the optimal combination of DNA methylation-dysregulated lncRNA biomarkers. Then a DNA methylation-dysregulated lncRNA signature (DNAMeLncSig) was developed by constructing a linear score model of expression levels of optimal DNA methylation-dysregulated lncRNA biomarkers, weighted by their estimated regression coefficients from the multivariate regression analysis as previous studies (Zhou et al., 2015a, b; Bao et al., 2021).

### Statistical Analysis

Hierarchical clustering analysis was carried out using the R package “pheatmap” with “ward.D2” method. The median risk score of the DNAMeFourLncSig was selected as a risk cutoff point to divide patients into the high-risk group (>cutoff) and low-risk group (≤cutoff). The Kaplan-Meier estimate was used to compare survival differences between the low-risk and high-risk groups, and statistical significance was examined using the log-rank test. To test whether DNA methylation-dysregulated lncRNA signature was independent of other clinical factors, univariate and multivariable Cox regression analysis and data stratification analysis were conducted. The time-dependent receiver operating characteristic (ROC) curves were used to compare the sensitivity and specificity of the 3- and 5-year survival prediction based on the DNA methylation-dysregulated lncRNA signature and the area under the curve (AUC) was calculated.

### *In silico* Analysis of Functional Roles of the DNA Methylation-Dysregulated lncRNA Signature

The PCC was calculated between expression levels of lncRNAs and mRNAs to identify the mRNAs (ranked top 10%) correlated with the DNA methylation-dysregulated lncRNA signature. The functional roles of the DNA methylation-dysregulated lncRNA signature were *in silico* predicted through function enrichment analysis of Gene Ontology (GO) and Kyoto Encyclopedia of Genes and Genomes (KEGG) using the R package “clusterProfiler” (Yu et al., 2012).

## Results

### Identification of DNA Methylation-Dysregulated Prognostic lncRNAs

**FIGURE 1:**
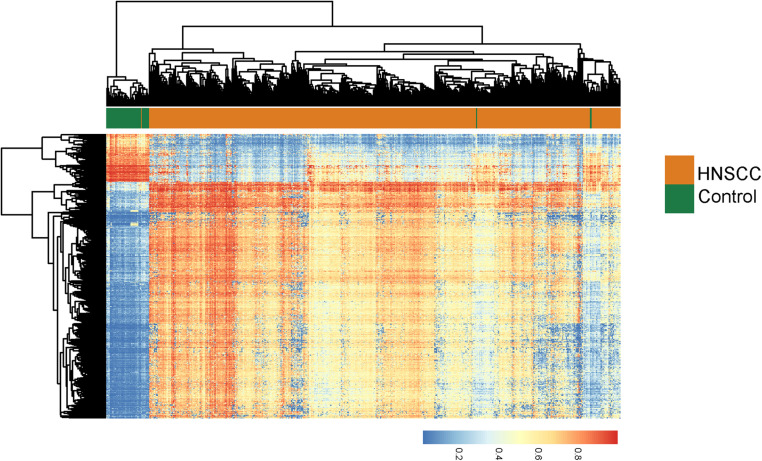
Unsupervised hierarchical clustering heatmap of 528 HNSCC tumor tissues and 50 normal tissues based on 780 differentially methylated sites.

We first performed differential DNA methylation analysis between 528 HNSC tumor tissues and 50 normal tissues using the R package “limma,” and identified 780 differentially methylated sites with FDR adjusted *p* < 0.05 and absolute mean methylation difference > 0.4. Hierarchical clustering of 780 differentially methylated sites separated HNSC tumor tissues from normal tissues (Figure 1). Then we measured the relationship between 780 differentially methylated sites and 14,618 lncRNA expression by calculating the PCC and found that expression levels of 596 lncRNAs are significantly correlated with these differentially methylated sites, which could be considered as DNA methylation-dysregulated lncRNAs. Finally, we conducted the training-validation study by randomly and equally dividing TCGA patients into discovery cohort (*n* = 250) and validation cohort (*n* = 249). We then performed univariate Cox proportional hazards regression for 596 DNA methylation-dysregulated lncRNAs with overall survival and found that 6 of 596 DNA methylation-dysregulated lncRNAs are significantly associated with overall survival and were considered as candidate prognostic biomarkers.

### Derivation of a DNA Methylation-Dysregulated lncRNA Signature From the Discovery Cohort

**FIGURE 2:**
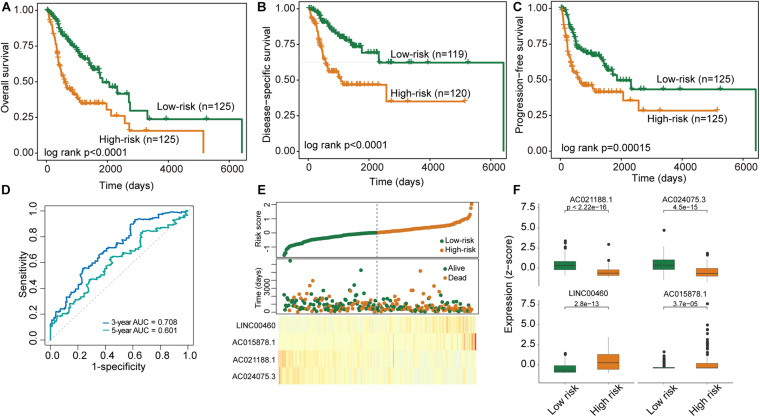
Performance evaluation of the DNAMeFourLncSig in the discovery cohort. **(A–C)** Kaplan-Meier survival curves of survival between low-risk and high-risk groups. **(D)** ROC analysis of the DNAMeFourLncSig at 3- and 5-years. **(E)** The distribution of DNAMeFourLncSig risk score, survival status and expression heatmap of patients. **(F)** Boxplots for expression levels of four lncRNA biomarkers between low-risk and high-risk groups.

To construct a clinically usable lncRNA signature, we performed feature selection using the stepwise regression method by successively adding or removing prognostic lncRNA biomarkers and identified four lncRNAs (*AC021188.1, AC024075.3, LINC00460*, and *AC015878.1*) as an optimal combination using the Akaike information criterion (AIC) (Table 1). Then these four optimal lncRNAs biomarkers were fitted into multivariate Cox regression analysis to obtain their relative power in survival prediction. Finally, a DNA methylation-dysregulated four-lncRNA signature (DNAMeFourLncSig) was developed as a linear scoring of lncRNA expression values weighted by coefficients derived from multivariate Cox regression analysis as follows: DNAMeFourLncSig = (-0.29216)^∗^expression of *AC021188.1* + (-0.24336)^∗^ expression of *AC024075.3* + (0.17071)^∗^expression of *LINC00460* + (0.30463)^∗^expression of *AC015878.1*. We then calculated the risk score based on the DNAMeFourLncSig for each patient in the discovery and ranked them according to their risk score. The median risk score (0.0127) of the DNAMeFourLncSig was selected as a risk cutoff point to divide patients into the high-risk group (>cutoff) and low-risk group (≤cutoff). As shown in Figure 2A, patients in the low-risk group had significantly improved overall survival than those in the high-risk group (median survival 1838 days vs. 606 days, log-rank *p* < 0.0001) (Figure 2A). The 3- and 5-year overall survival rates of patients in the low-risk group are 68 and 53%, respectively, higher than corresponding rates (37 and 35%). Furthermore, disease-specific and progression-free survival time of the low-risk group patients was significantly longer than those of high-risk group patients (median disease-specific survival 6,417 days vs. > 60 months, log-rank *p* < 0.0001, and median progression-free survival 1,859 days vs. 614 days, log-rank *p* = 0.00015) (Figures 2B,C). ROC analysis found that the DNAMeFourLncSig achieved an AUC value of 0.708 and 0.601 in survival prediction at 3 and 5 years (Figure 2D). The distribution of DNAMeFourLncSig risk score, survival status and expression heatmap of patients in the discovery cohort was shown in Figure 2E. Two lncRNAs (*LINC00460* and *AC015878.1*) were found to have higher expression in a high-risk group that is significantly associated with poor overall survival (HR = 1.26, 95% CI = 1.07–1.48, p = 0.0056 for *LINC00460*, and HR = 1.26, 95% CI = 1.08–1.46, *p* = 0.003 for *AC015878.1*). Other two lncRNAs (*AC021188.1* and *AC024075.3*) were over-expressed in the low-risk group that are significantly associated with proved overall survival (HR = 0.71, 95% CI = 0.57–0.89, *p* = 0.003 for *AC021188.1*, and HR = 0.76, 95% CI = 0.62–0.93, *p* = 0.0072 for *AC024075.3*).

**TABLE 1:** Detailed information of four prognostic lncRNA biomarkers.

Ensembl ID	Gene ID	Chromosomal location	HR	95%CI	*P*-value
ENSG00000230747	AC021188.1	Chr2: 96,307,263–96,321,731(−)	0.71	0.57–0.89	0.003
ENSG00000269427	AC024075.3	Chr 19: 16,630,743–16,643,942(+)	0.76	0.62–0.93	0.0072
ENSG00000233532	LINC00460	Chr 13: 106,374,477–106,384,315(+)	1.26	1.07–1.48	0.0056
ENSG00000265751	AC015878.1	Chr 18: 21,380,286–21,451,017(−)	1.26	1.08–1.46	0.003

### Independent Validation of the DNAMeFourLncSig in the Validation Cohort

**FIGURE 3:**
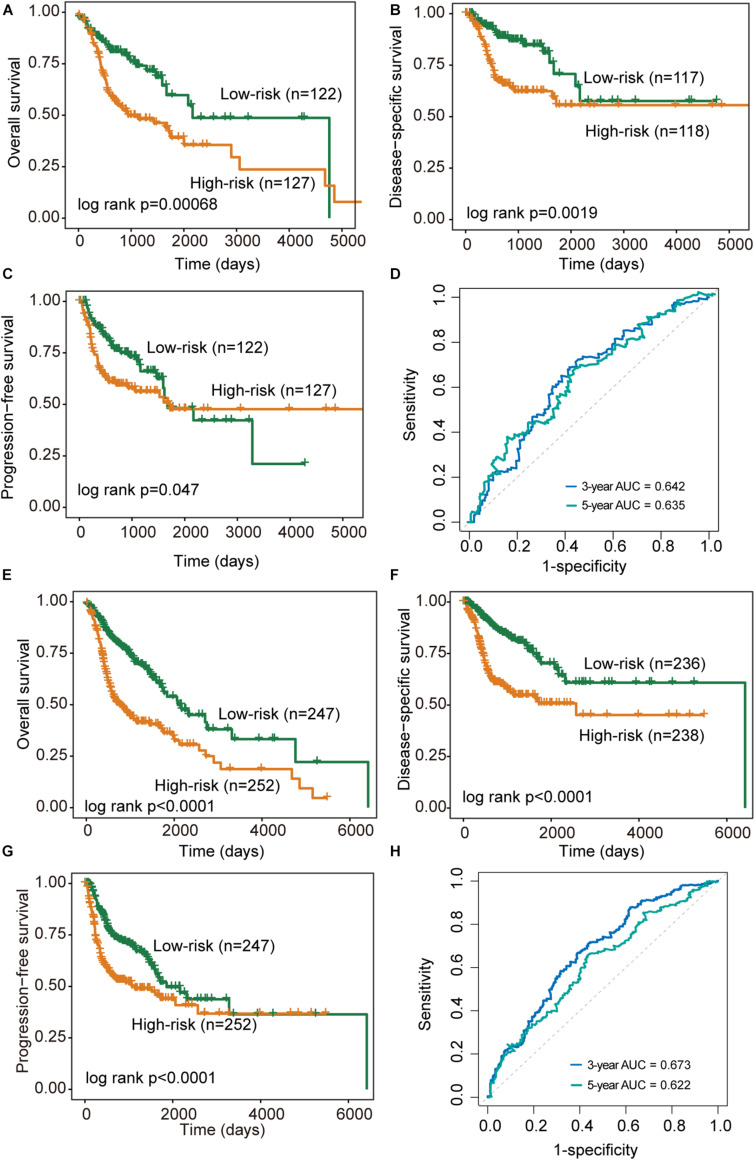
Performance validation of the DNAMeFourLncSig. **(A–C)** Kaplan-Meier survival curves of survival between low-risk and high-risk groups in the validation cohort. **(D)** ROC analysis of the DNAMeFourLncSig at 3- and 5-years in the validation cohort. **(E–G)** Kaplan-Meier survival curves of survival between low-risk and high-risk groups in the TCGA cohort. **(H)** ROC analysis of the DNAMeFourLncSig at 3- and 5-years in the TCGA cohort.

We further validated the DNAMeFourLncSig in an independent validation cohort to examine the robustness and reliability of the DNAMeFourLncSig in prognosis prediction. The same cutoff point from the discovery cohort was used to separate patients in the validation cohort into the high-risk group (*n* = 127) and low-risk group (*n* = 122). There was a significantly different prognosis between the high-risk group and the low-risk group. As shown in Figure 3A, the overall survival time of patients in the high-risk group was significantly shorter than that of the patient in the low-risk group (median survival 941 days vs. 2,166 days, log-rank *p* = 0.00068). The 3- and 5-year overall survival rates of patients in the low-risk group are 74 and 60%, respectively, higher than corresponding rates (50 and 39%). Similar differences also were observed for disease-specific survival (log-rank *p* = 0.0019) and progression-free survival (median survival 1671 days vs. 1,718 days, log-rank *p* = 0.047) (Figures 3B,C). The DNAMeFourLncSig achieved an AUC value of 0.642 and 0.635 in survival prediction at 3 and 5 years (Figure 3D).

When the DNAMeFourLncSig was further tested in the entire TCGA cohort, all patients were classified as high-risk (*n* = 252) and low-risk (*n* = 247) according to their DNAMeFourLncSig. The two groups of patients differ significantly in overall survival (median survival 2,083 days vs. 763 days, log-rank *p* < 0.0001), disease-specific survival (median survival 6,417 days vs. 2,570 days, log-rank *p* < 0.0001) and progression-free survival (median survival 1,859 days vs. 1,064 days, log-rank *p* < 0.0001) (Figures 3E–G). Furthermore, DNAMeFourLncSig achieved an AUC value of 0.673 and 0.622 in survival prediction at 3 and 5 years (Figure 3H).

### Independence of the DNAMeFourLncSig of Other Clinical Features

The results of univariate Cox regression analysis revealed that the DNAMeFourLncSig and treatment response are all significantly associated with overall survival in all three cohorts, as shown in Table 2. Therefore we further investigated whether the prognostic value of DNAMeFourLncSig was independent of other clinicopathological factors and treatment response using multivariate Cox regression. Results of multivariate analysis from the discovery cohort showed that the DNAMeFourLncSig (HR = 2.28, 95% CI = 1.31–3.97, *p* = 0.0038) and treatment response (HR = 0.25, 95% CI = 0.13–0.47, *p* = 2.4e-05) still maintained a significant association with overall survival after adjusted by other clinicopathological factors (Table 2). Similar associations from multivariate analysis also were observed in the validation cohort and TCGA cohort (Table 2). These results indicated that the DNAMeFourLncSig might be an independent prognostic factor in predicting survival.

**TABLE 2:** Univariable and multivariable Cox regression analyses in each patient cohort.

Variables		Univariate analysis			Multivariate analysis		
		HR	95%CI	*p*-value	HR	95%CI	*p*-value
**Discovery cohort**							
DNAMeFourLncSig	High vs. Low	2.45	1.69–3.56	2.3e−06	2.28	1.31–3.97	0.0038
Age	>60 vs. =60	1.05	0.73–1.5	0.8	1.13	0.7–1.83	0.62
Stage	III/IV vs. I/II	1	0.63–1.57	0.99	1.07	0.58–1.95	0.84
Grade	(III/IV vs. I/II)	1.08	0.73–1.62	0.69	1.65	0.96–2.84	0.068
Treatment response	CR vs. non-CR	0.16	0.09–0.29	6.6e−10	0.25	0.13–0.47	2.4e−05
**Validation cohort**							
DNAMeFourLncSig	High vs. Low	2.06	1.35–3.16	0.00089	3.21	1.72–5.97	0.00024
Age	>60 vs. =60	1.51	1–2.28	0.049	2.13	1.2–3.79	0.01
Stage	III/IV vs. I/II	1.41	0.88–2.26	0.15	1.74	0.87–3.5	0.12
Grade	(III/IV vs. I/II)	0.79	0.51–1.23	0.3	1.07	0.59–1.96	0.82
Treatment response	CR vs. non-CR	0.2	0.1–0.4	3.8e−06	0.27	0.14–0.53	0.00015
**TCGA cohort**							
DNAMeFourLncSig	High vs. Low	2.22	1.68–2.94	2e–08	2.53	1.69–3.79	5.9e−06
Age	>60 vs. =60	1.23	0.94–1.61	0.14	1.49	1.04–2.13	0.03
Stage	III/IV vs. I/II	1.22	0.88–1.69	0.23	1.43	0.91–2.25	0.12
Grade	(III/IV vs. I/II)	0.9	0.67–1.22	0.5	1.26	0.85–1.88	0.25
Treatment response	CR vs. non-CR	0.18	0.11–0.27	8e–15	0.24	0.15–0.37	5.9e−10

### Association of the DNAMeFourLncSig With Treatment Response

**FIGURE 4:**
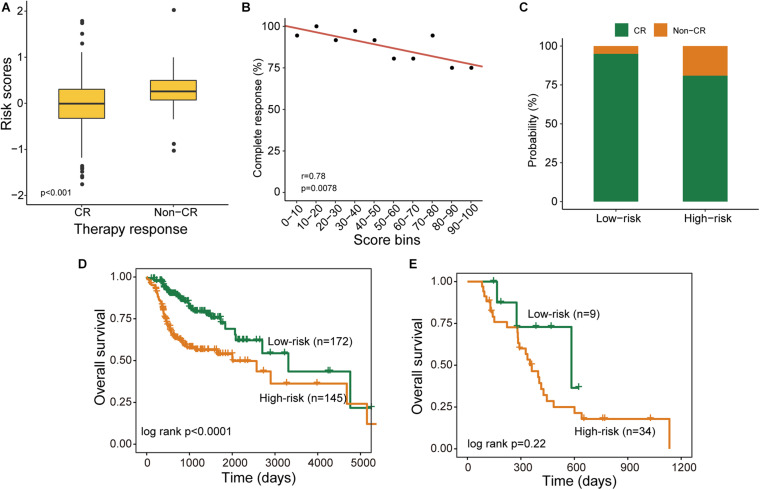
Association of the DNAMeFourLncSig with treatment response. **(A)** Boxplots for DNAMeFourLncSig risk score in patients with and without complete response. **(B)** Correlation between DNAMeFourLncSig risk score and complete response rate. **(C)** Boxplots for the probability of patients with and without complete response in the low-risk and high-risk groups. Kaplan-Meier survival curves of survival between low-risk and high-risk groups for CR patients **(D)** and non-CR patients **(E)**.

As shown in Table 2, treatment response is also a prognostic factor in the univariate and multivariate analysis. Therefore, we further examined the association of the DNAMeFourLncSig with treatment response. By comparing the distribution of DNAMeFourLncSig risk score, we found that DNAMeFourLncSig risk scores in patients achieving complete response (CR) are significantly lower than those in those achieving no complete response (non-CR) (Wilcoxon rank-sum test *p* < 0.001) (Figure 4A). Moreover, there is a significantly negative correlation between the DNAMeFourLncSig with CR rate (Pearson correlation *r* = 0.78, *p* = 0.00791) (Figure 4B). Patients achieving CR were enriched in the low-risk group and those non-CR patients were enriched in the high-risk group (Figure 4C). For patients with CR, the DNAMeFourLncSig still stratified patients into the high-risk and low-risk group with significantly different survival (median survival 2,002 days vs. 3,314 days, log-rank *p* < 0.0001) (Figure 4D). For non-CR patients, although it is no significant difference in survival between high-risk and risk groups, it still could be observed that non-CR patients in the low group have more prolonged survival than those in the high-risk group (median survival 584 days vs. 361 days) (Figure 4E).

### Functional Analysis of the DNAMeFourLncSig

**FIGURE 5:**
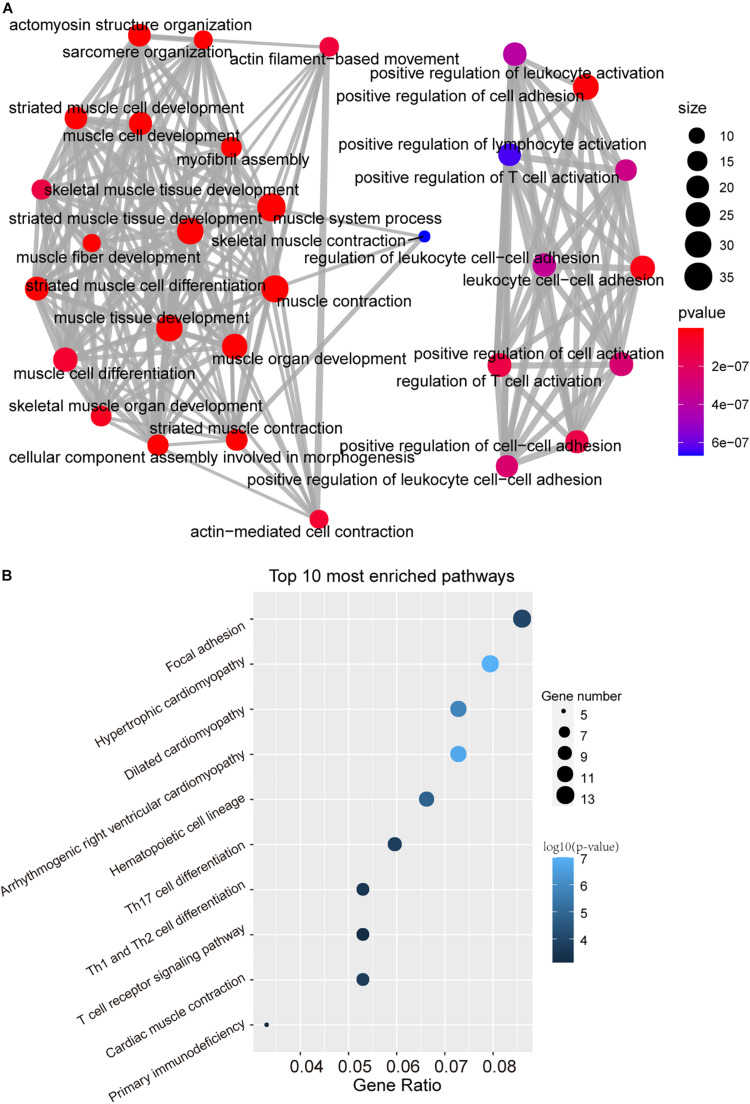
Function *in silico* analysis of the DNAMeFourLncSig. **(A)** The network of enriched GO terms. **(B)** Enriched KEGG pathways.

To further investigate the functional roles of the DNAMeFourLncSig in HNSC, we first evaluated the correlation between lncRNAs in the DNAMeFourLncSig and mRNAs by calculating the PCC and identified 400 mRNAs as DNAMeFourLncSig-related mRNAs. Then we performed KEGG and GO enrichment analysis for these DNAMeFourLncSig-related mRNAs, and found that DNAMeFourLncSig-related mRNAs were mainly enriched in cell differentiation, tissue development and immune-related pathways (Figures 5A,B).

## Discussion

HNSCC is an aggressive, life-threatening disease associated with low survival rates. Although traditional tumor-node-metastasis (TNM) staging system in combination with some risk factors, such as exposure to environmental pollutants and infection with viral agents, tobacco and alcohol consumption, have widely been used in current clinical practice, early diagnosis and prognosis prediction remains highly challenging (Hsieh et al., 2019; Johnson et al., 2020). With the advance in high-throughput omics technology, improved understanding of molecular mechanisms of HNSCC has revealed molecular heterogeneity associated with different behaviors (Zhang et al., 2014; Leemans et al., 2018), implying the potential for molecular changes as novel biomarkers to additional information relevant to TNM staging.

Aberrant expression of lncRNAs has been observed in various cancers, including HNSCC (Zou et al., 2016; Guo et al., 2018; Ghafouri-Fard et al., 2020). However, epigenetically dysregulated lncRNAs and their functional and clinical roles in HNSCC are still not explored. In this study, we performed an integrative analysis of DNA methylation data and transcriptome data and identified 596 DNA methylation-dysregulated lncRNAs. Of them, six DNA methylation-dysregulated lncRNAs were significantly associated with patient outcomes. Therefore, we conducted a machine-learning feature selection analysis using a stepwise regression method to search for an optimal lncRNA combination from six prognostic DNA methylation-dysregulated lncRNAs. As a result, an optimal lncRNA combination consisting of four prognostic DNA methylation-dysregulated lncRNAs (*AC021188.1, AC024075.3, LINC00460*, and *AC015878.1*) was identified and subsequently were transformed into a scoring model (named DNAMeFourLncSig), which classified the patients of the discovery cohort into two risk groups with significantly different survival including overall survival, disease-specific survival, and progression-free survival. To examine the reliability and robustness of the DNAMeFourLncSig, we tested this DNAMeFourLncSig in the other two patient cohorts, which showed similar prognostic values in both. Results from multivariable Cox regression analysis indicated that the DNAMeFourLncSig is an independent prognostic factor. Furthermore, the DNAMeFourLncSig was significantly correlated with the complete response rate of chemotherapy and may predict chemotherapy response.

Of four lncRNA biomarkers in the DNAMeFourLncSig, the dysregulated expression of LINC00460 has recently been reported to affect cell proliferation and apoptosis and are closely associated with cancer development and metastasis. By comparing *LINC00460* expression in 92 pairs of colorectal cancer and adjacent normal tissues, Wang et al. found that upregulated LINC00460 expression was associated with early-stage CRC and low disease-free survival (Wang et al., 2018). Further study *In vitro* and *in vivo* assays by Lian found that *LINC00460* function as ceRNA to contribute to CRC tumorigenesis and progression by Regulating *KLF2* and *CUL4A* Expression (Lian et al., 2018). Moreover, Jiang’s study *in vitro* and *in vivo* provided direct evidence supporting the association of the *LINC00460* and HNSC progression. They found that *LINC00460* enhanced HNSCC cell proliferation and metastasis by promoted EMT in HNSCC cells by facilitating *PRDX1* entry into the nucleus to induce epithelial-mesenchymal transition (Jiang Y. et al., 2019). Another lncRNAs, *AC021188.1*, has also been found to be associated with prognosis and was included in a 5-disease prognostic signature lncRNAs in HNSCC in Liu’s study (Liu et al., 2018). It has been shown that lncRNA function could be inferred by studying the functional roles of lncRNA-related mRNAs (Liao et al., 2011; Zhou et al., 2018, 2019). As described in other studies, we first measured co-expression relationships between lncRNA biomarkers and mRNAs to identify DNAMeFourLncSig-related mRNAs. Then we performed functional enrichment analysis for these DNAMeFourLncSig-related mRNAs to identify over-represented GO terms and KEGG pathways. *In silico* functional analysis demonstrated that DNAMeFourLncSig might participate in cell differentiation, tissue development and immune-related pathways.

Although our study identified and validated this DNA methylation-dysregulated four-lncRNA signature for predicting the prognosis of patients with HNSCC, several limitations should be acknowledged. Firstly, the DNAMeFourLncSig should need to be further tested in other patient cohorts to confirm its possibility for clinical application. Second, although two lncRNAs of the DNAMeFourLncSig have been functionally studied in previous reports, the functional roles of the other two lncRNA should be studied through an experimental approach.

## Data Availability Statement

Publicly available datasets were analyzed in this study. This data can be found here: Clinical information, DNA methylation data and RNA-seq data of HNSCC patients were downloaded from were retrieved from the UCSC Xena Browser: cohort GDC TCGA Head and Neck Cancer (HNSC) (https://xena.ucsc.edu/).

## Author Contributions

TW and MZ conceived, designed the experiments, and wrote the manuscript. QW, WY, WP, and XQ analyzed the data. All authors read and approved the final manuscript.

## Conflict of Interest

The authors declare that the research was conducted in the absence of any commercial or financial relationships that could be construed as a potential conflict of interest.

## Footnotes

1 https://xena.ucsc.edu/

2 https://www.gencodegenes.org/
